# How do people with dementia present to the services, and why do they present late? A descriptive study in a Tertiary Care Hospital in Sri Lanka

**DOI:** 10.1192/bjo.2021.677

**Published:** 2021-06-18

**Authors:** Malsha Gunathilake, Chathurie Suraweera

**Affiliations:** 1 North East London Foundation Trust; 2 Faculty of Medicine, University of Colombo

## Abstract

**Aims:**

To assess how patients with dementia present to services and reasons for delayed presentation among patients with dementia in Sri Lanka.

**Method:**

A descriptive cross-sectional study was conducted among 83 newly diagnosed patients with dementia and their caregivers at the University Psychiatry Unit, National Hospital of Sri Lanka. They were interviewed using a semi-structured pre-tested questionnaire. Statistical Package for the Social Sciences (SPSS) was utilized for data analysis.

**Result:**

The mean age of the patients was 71.53(SD = 7.595)years. The commonest type of dementia in the cohort was Alzheimer's disease(N = 49, 59%). The mean untreated duration before the first presentation was 16.33(SD = 16.13) months. A family member or the care-giver had initiated help-seeking in many (N = 65,78.3%). 84.33% of patients had behavioural and Psychological Symptoms of Dementia (BPSD) at first presentation. BPSD was the main reason for help-seeking in 40(48.2%) cases. Among them, psychosis(n = 18,45%), depression(n = 9,22.5%), disinhibition(n = 4,10%) and wandering(n = 3,7.5%) were common.

Lack of awareness on dementia (n = 70,93.3% and n = 68,86.1%) and considering cognitive impairment as a normal part of ageing (n = 39,52% and n = 43,54.4%)were the commonest reasons for delayed presentation reported by patients and care-givers respectively. Twelve patients misattributed the symptoms to their existing medical or psychiatric conditions. The mean untreated duration was significantly higher in the patient group with a family history of dementia (30.5 months) compared to those without a family history (12.8 months)(t = 3.818;p = 0.000). Similarly, the mean untreated duration was significantly higher when there is a family history of dementia among the care-givers (25.53months) compared to the group of care-givers without a family history (13.85 months)(t = 2.532;p = 0.013). Age, sex, education, occupation, income, knowledge on dementia of the patients and the caregivers, illness-related characteristics (type, severity, and presence of BPSD) or being in contact with medical services were not significantly associated with the timing of the first presentation.

**Conclusion:**

There is a delay of more than one year for patients with dementia to present to services in Sri Lanka. The commonest reason for the presentation is BPSD. Lack of prior awareness of dementia and considering the cognitive impairment as a part of normal ageing by both patients and carers were the main reasons for delayed presentation. Patients with a family history of dementia present late than those without a family history. There is no significant association between the timing of presentation and the socio-demographic factors of the patients and care-givers, the presence of prior knowledge on dementia, illness-related characteristics, or contact with medical services.

